# A Physician's Nightmare: Fever of Unknown Origin

**DOI:** 10.1155/2016/5437971

**Published:** 2016-06-28

**Authors:** Sana Din, Farrukh Anwer, Mirza Beg

**Affiliations:** ^1^Upstate Medical University, Syracuse, NY 13210, USA; ^2^Auburn Community Hospital, Auburn, NY 13021, USA; ^3^Division of Pediatric Gastroenterology, Hepatology & Nutrition, Upstate Medical University, Golisano Children's Hospital, Syracuse, NY 13210, USA

## Abstract

Fever of unknown origin (FUO) remains to be a challenge despite advancement in diagnostic technologies and procedures. FUO is considered when fever presents intermittently without an explanation. It has been linked to various etiologies, which makes it difficult to diagnose. We present the case of 18-month-old female with recurrent fever, splenomegaly, abdominal pain, and constipation. The workup for her symptoms revealed wandering spleen. Wandering spleen is a result from excessive laxity or absence of splenic ligaments. The patient underwent splenectomy and was advised to continue on Senna, Miralax, and high fiber diet. Her mother reported that the fever is no longer present and there is marked improvement in her constipation and abdominal pain after splenectomy.

## 1. Introduction

A fever presenting in children is one of the most prevalent reasons for visiting family practitioners/pediatricians. In most cases, the fever is due to infectious cause and can subside within 10 days. When the fever does not subside within expected disease course, then the fever of unknown origin needs to be investigated. Fever of unknown origin [FUO] is intermittent high fever of more than 38-39° Celsius without clear known cause or symptoms, lasting for more than 8 days, more than 3 weeks in an outpatient setting, or more than 1 week in inpatient setting even with evaluation [1–3].

The spleen is a lymphoid organ located in left upper quadrant suspended by gastrosplenic and splenorenal ligaments [4]. Wandering spleen is a rare disorder that results from spleen being attached to pedicle instead of ligaments due to absence, abnormality, or laxity of the ligaments. The pedicle contains blood vessels and is longer than normal. If it is not fixed it can twist on itself easily [4, 5]. Wandering spleen is present in pediatric population with male to female ratio of 6 to 1.2 and females of childbearing age (hormones causing laxity of ligaments) [4]. The common causes of fever in relation to spleen are hemolytic anemia (sickle cell anemia, spherocytosis, and thrombotic thrombocytopenic purpura), infection (parvovirus, tuberculosis, infective endocarditis, cytomegalovirus, Epstein-Barr virus, brucellosis, rocky mountain spotted fever, typhoid fever, histoplasmosis, and malaria), sarcoidosis, leukemia, and lymphoma [1, 6, 7].

## 2. Case Presentation

18-month-old Caucasian female presented with recurrent fever that persisted for 2 weeks. Fever did not respond to Motrin or Tylenol. Her prenatal course and delivery were uncomplicated. Patient has a history of grade 3 vesicoureteral reflux, febrile seizure, and urinary tract infection (average of 1-2/year). Fever originally was thought to be due to UTI since patient has vesicoureteral reflux but urine analysis was negative for any treatment. Patient was on Bactrim prophylactically for vesicoureteral reflux. She had splenomegaly on physical exam and abdominal ultrasound confirmed it. Patient's CBC, WBC with differential, ESR, CMP, BUN/creatinine ratio, glucose, uric acid, lactate dehydrogenase, electrolytes, and liver function enzymes were normal. Patient had elevated CRP, which indicated that something is going on with the child even though we could not find any obvious cause. She was negative for rheumatoid factor, EBV titer,* Bartonella* titers, CMV titers, HIV antibody,* Toxocara*,* Blastomyces* antibody, and* Histoplasma* antibodies. Patient has normal immunoglobulins, B cells, T cells, and CD4 levels. The patient's workup for hematology/oncology and rheumatology was negative. Patient did not show any evidence of storage disease. She had no evidence of neurologic or hematologic dysfunction. An ultrasound of the abdomen with Doppler was done because of splenomegaly and fever. Ultrasound showed splenomegaly and irregular spleen that is located in left lower quadrant. Doppler investigation showed normal blood flow in hepatic vein, portal vein, and splenic vein in midline. We continued to investigate the cause of recurrent fevers, abdominal pain, and splenomegaly via CT scan of the abdomen and pelvis. The CT scan showed enlarged spleen and ill-defined foci of hypoattenuation with the right kidney. She was given the diagnosis of wandering spleen based on ultrasound and CT scan images. After discussion with parents, laparoscopic splenectomy was performed without any complication and Penicillin VK was prescribed prophylactically to prevent sepsis. After surgery, patient did not experience recurrent fever and her abdominal pain improved significantly.

## 3. Discussion

Peterssdorf and Beeson defined FUO in 1961 as presence of fever of more than 38° Celsius in more than one occasion, presence of fever for more than 3 weeks, and lastly failure to reach a definitive diagnosis despite inpatient evaluation [8, 9]. FUO is considered when fever is present for about 5–21 days along with medical evaluation [1]. FUO can cause increased morbidity if the diagnosis is missed of a serious illness or an easily treatable cause. Fever can be caused by infection (bacterial, fungi, and viruses), oncologic disease (leukemia and lymphoma), noninfectious inflammatory/autoimmune disease (Crohn's disease, sarcoidosis, and systemic lupus erythematous), vasculitis syndrome (polyarteritis nodosa and Kawasaki disease), genetic disease, drugs (anticonvulsants, antihistamines, antimicrobials, cardiovascular drugs, adrenal insufficiency medication, nonsteroidal anti-inflammatory medication), factitious fever, lysosomal storage diseases (Fabry and Niemann-Pick disease), iatrogenesis, and thyroiditis [1, 2]. Pseudo-FUO is consecutive incidences of infectious illnesses accompanied by fever that can be perceived as one extended episode of fever present for longer period by parents. Pseudo-FUO presents initially as clear diagnosis of an infection that resolves but is later followed by another febrile infection. Pseudo-FUO needs to be ruled out before making a definitive diagnosis of FUO to avoid extensive and unnecessary evaluation [1]. Diagnosis of pseudo-FUO requires detailed history documenting afebrile and febrile episodes [1].

The initial step in diagnosing FUO is to document the presence of fever and timing of administration of antipyretic. It is important to determine the pattern (intermittent, recurrent, remittent, and sustained), frequency, and timing of fever. FUO is usually accompanied by symptoms; therefore, it is very important to have detailed review of systems because the onset of symptoms and the timing of the fever can lead to a possible diagnosis [1]. Also, it is crucial to take repeated history and encourage parents/patient to report any new signs or symptoms. Another important step in evaluation of FUO is serial physical examinations in a controlled setting to document vital signs and weight loss. Initial workup in a well appearing child with fever for more than 8 days is to stop unnecessary medications and to do the following analyses: CBC with differentials, basic metabolic panel, liver function tests, urinalysis, radiographs as indicated, CSF studies if there are neurological symptoms, and observation [1]. Admit the patient if ill appearing and start initial workup (stop unnecessary medications, do the following analyses: CBC with differentials, basic metabolic panel, liver function tests, C-reactive protein, urinalysis, radiographs as indicated, CSF studies if there are neurological symptoms, start antibiotics after taking cultures, and start serial physical examinations) [1]. Further evaluation should be targeted toward any positive finding in initial laboratory workup. If the source is identified or fever resolves in both well and ill appearing patient then treat the source leading to the presentation. As seen in our patient, the initial and targeted workup did not identify the source; therefore, further workup was needed in oncologic, autoimmune, rheumatology, infectious, and immunodeficiency cases [1]. Mortality rate in pediatric FUO was of 6–9% in 1970 but unknown for current FUO cases [1]. As in our patient, the history, physical exam, and diagnostic investigation showing splenomegaly are a potential diagnostic clue towards spleen related etiology for FUO. Splenomegaly can be caused by leukemia, lymphoma, infections, and wandering spleen [4].

Spleen filters blood and stores white blood cells and platelets. It is responsible for tissue inflammation and immune response [10]. Spleen is composed of 2 types of tissues, the red pulp and the white pulp. Red pulp is involved in filtration of red blood cells and it is composed of sinuses (has RBC) and splenic cords. Marginal zone represents a border between red pulp and white pulp. White pulp is involved in active immune response through humoral or cell mediated immunity and it is composed of germinal centers (produces lymphocyte), lymphoid follicles (has B cells), and periarteriolar lymphoid sheath (has T cells) [10]. Spleen responds to foreign bodies and antigens present in blood by releasing phagocytes and by production of antibodies [10]. For example, if the patient is infected with pneumococcus, the immune response takes place when the circulating antigens enter the marginal zone. Fever results from release of cytokines in an active immune response. The polysaccharide is presented to B cells which release IGg antibodies. IGg activates compliment system and opsonization to prepare membrane attack complex resulting in lyses of pneumococcus [11]. Our patient was on Penicillin prophylaxis after splenectomy to prevent sepsis. Wandering spleen has rarely been associated with FUO. The incidence of wandering spleen is around 5% known from series of splenectomies and less than 100 reported cases in pediatrics [4]. Wandering spleen is a rare condition due to lack of ligamentous attachment of a spleen to diaphragm, colon, and retroperitoneum. This results in various position of spleen in the abdomen due to laxity, mobility, and rearrangements [9]. The spleen can twist around the long pedicle leading to ischemia and necrosis. This can present with severe abdominal pain, vomiting, and fever due to splenic torsion. Our patient did not experience ischemia and necrosis leading to acute abdomen. In symptomatic patients, 50% of mortality rate is due to splenic torsion because infarction, rupture, or abscess formation requires emergent splenectomy [4]. After splenectomy, patients are at increased risk of developing sepsis from an encapsulated organism; therefore Penicillin prophylaxis was prescribed to our patient. Wandering spleen can also present as a chronic, intermittent mobile abdominal mass that is painful with any movement except in left upper quadrant [12, 13]. The preferred treatment for asymptomatic patient is splenopexy [4]. Early diagnosis is crucial to preserve the spleen and to avoid development of further complications [12]. Wandering spleen is linked with infectious mononucleosis, splenomegaly, malaria, Hodgkin's disease, pregnancy, and postsurgical correction of gastrosplenic ligament [13]. As seen with our patient, wandering spleen can be confirmed with ultrasound showing splenomegaly, absence of spleen in left hypochondrium, or low position of spleen and by contrast enhanced CT showing whorl sign [4].

## 4. Conclusion

FUO can be linked to various causes, ranging from infections to noninfectious etiology, and can be a benign disease or severe disease. Diagnosis requires detailed evaluation, procedures, and cooperation between healthcare providers. We presented this case to emphasize that wandering spleen should be in differential diagnosis for FUO. This will help in early diagnosis, treatment, preserving spleen, and patient satisfaction.
